# APOBEC signature mutation generates an oncogenic enhancer that drives *LMO1* expression in T-ALL

**DOI:** 10.1038/leu.2017.75

**Published:** 2017-03-28

**Authors:** Z Li, B J Abraham, A Berezovskaya, N Farah, Y Liu, T Leon, A Fielding, S H Tan, T Sanda, A S Weintraub, B Li, S Shen, J Zhang, M R Mansour, R A Young, A T Look

**Affiliations:** 1 Department of Pediatric Oncology, Dana-Farber Cancer Institute, Harvard Medical School, Boston, MA, USA; 2 Whitehead Institute for Biomedical Research, Cambridge, MA, USA; 3 Department of Haematology, UCL Cancer Institute, University College London, London, UK; 4 Department of Computational Biology, St Jude Children’s Research Hospital, Memphis, TN, USA; 5 Cancer Science Institute of Singapore, National University of Singapore, Singapore, Singapore; 6 Department of Medicine, Yong Loo Lin School of Medicine, Singapore, Singapore; 7 Department of Hematology and Oncology, Key Laboratory of Pediatric Hematology and Oncology Ministry of Health, Shanghai Children’s Medical Center, Shanghai Jiao Tong University School of Medicine, Shanghai, China; 8 Pediatric Translational Medicine Institute, Shanghai Jiao Tong University School of Medicine, Shanghai, China; 9 Department of Biology, Massachusetts Institute of Technology, Cambridge, MA, USA; 10 Division of Hematology/Oncology, Children’s Hospital, Boston, MA, USA

## Abstract

Oncogenic driver mutations are those that provide a proliferative or survival advantage to neoplastic cells, resulting in clonal selection. Although most cancer-causing mutations have been detected in the protein-coding regions of the cancer genome; driver mutations have recently also been discovered within noncoding genomic sequences. Thus, a current challenge is to gain precise understanding of how these unique genomic elements function in cancer pathogenesis, while clarifying mechanisms of gene regulation and identifying new targets for therapeutic intervention. Here we report a C-to-T single nucleotide transition that occurs as a somatic mutation in noncoding sequences 4 kb upstream of the transcriptional start site of the *LMO1* oncogene in primary samples from patients with T-cell acute lymphoblastic leukaemia. This single nucleotide alteration conforms to an APOBEC-like cytidine deaminase mutational signature, and generates a new binding site for the MYB transcription factor, leading to the formation of an aberrant transcriptional enhancer complex that drives high levels of expression of the *LMO1* oncogene. Since APOBEC-signature mutations are common in a broad spectrum of human cancers, we suggest that noncoding nucleotide transitions such as the one described here may activate potent oncogenic enhancers not only in T-lymphoid cells but in other cell lineages as well.

## Introduction

Despite enormous efforts expended on the resequencing of human tumour genomes over the past decade, almost all of such efforts have focused on the discovery of coding mutations.^1^ Many critical proto-oncogenes become oncogenic due to aberrant overexpression in human cancer cells through genomic abnormalities such as chromosomal translocations, inversions and deletions in noncoding genomic sequences.^2^ Recent discoveries of small scale mutations in noncoding gene regulatory regions have inspired considerable interest in identifying abnormalities that create strong transcriptional enhancers or promoters capable of driving the expression of critical oncogenes in human cancer.^3, 4, 5, 6^ Although such efforts to identify 'driver mutations' in the noncoding genome and distinguish them from 'passengers' has been difficult, it could be important as a way to implicate targetable oncogenes for 'precision medicine', whose overexpression is based on clonal selection for aberrant transcriptional enhancers.

The LIM-domain-only (LMO) proteins (LMO1-4) are transcriptional regulatory proteins that are not able to directly bind to DNA but rather contain two LIM domains that mediate protein-protein interactions.^7, 8, 9, 10^ In T-cell acute lymphoblastic leukaemia (T-ALL) cells, either LMO1 or LMO2 is a critical component of a transcriptional complex comprised of TAL1, TCF12/HEB, TCF3/E2A, MYB, RUNX1, GATA3 and LDB1, which forms a positive interconnected auto-regulatory circuit that is a major driver of malignant transformation in ~60% of cases of T-ALL in children and adults.^5, 10, 11, 12^

Both LMO1 and LMO2 are downregulated as thymocytes progress in differentiation to the double-positive stage,^10, 13, 14^ and a critical event in transformation in this genetic subtype of T-ALL is the aberrant upregulation of one of these two genes. One cause of aberrant expression of LMO1 is the t(11;14)(p15;q11) *LMO1-TCRD* rearrangement.^9, 15, 16, 17^ However, <1% of T-ALL patients harbour activating translocations involving *LMO1,* which cannot explain its overexpression in each T-ALL case that aberrantly overexpresses the *LMO1* mRNA,^18, 19, 20^ suggesting that other types of genetic abnormalities can cause aberrant expression of the *LMO1* gene.

## Materials and methods

### Human T-ALL cell lines

The identities of T-ALL cell lines were confirmed by analysis of short tandem repeats using the PowerPlex 1.2 system (Promega, Fitchburg, WI, USA) in January 2013, and the T-ALL cell lines used for ChIP-seq in this study were reconfirmed in February 2016. All T-ALL cell lines were cultured in RPMI-1640 medium supplemented with 10% FBS, L-glutamine and penicillin/streptomycin (Invitrogen, Waltham, MA, USA). HEK-293 T cells were maintained in Dulbecco’s modified Eagle’s medium supplemented with 10% FBS, L-glutamine and penicillin/streptomycin. Cell lines were tested for mycoplasma contamination and found negative before used for experiments.

### Sequencing of the LMO1 enhancer region in T-ALL cell lines

The 739-bp genomic region of the *LMO1* enhancer was amplified by PCR using Phusion High-Fidelity DNA polymerase (New England Biolabs, Inc., Ipswich, MA, USA). The primers used are 5′-CACTTCGTCCTTCAGGCACT-3′ and 5′-CGGCGGGATTAGGAAGTCTC-3′. PCR products were purified using QIAquick PCR purification kit (Qiagen, Venlo, The Netherlands) and sent for Sanger sequencing in both forward and reverse orientation (Genewiz, Inc., Cambridge, MA, USA).

### Quantitative reverse transcriptase PCR (qRT-PCR)

RNeasy kit (Qiagen) was used to harvest total RNA from T-ALL cells, which was then reverse transcribed with Superscript III (Invitrogen). Quantitative PCR analysis was conducted with the ViiATM 7 system (Life Technologies, Waltham, MA, USA) using SYBR Green PCR Master Mix (Roche, Basel, Switzerland) and the following specific primers sets for each gene: LMO1-F: 5′-CGCAAGATCAAGGACCGCTA-3′; LMO1-R: 5′-GCATCACCATCTCGAAGGCT-3′ LMO2-F: 5′-TCGGCCATCGAAAGGAAGAG-3′; LMO2-R: 5′-ATGGCCTTCAGGAAGTAGCG-3′; 18S-F: 5′-AACCCGTTGAACCCCATT-3′; 18S-R: 5′-CCATCCAATCGGTAGTAGCG-3′; MYB-F: 5′-TGTTGCATGGATCCTGTGTT-3′ MYB-R: 5′-AGTTCAGTGCTGGCCATCTT-3′.

### Analysis of SNPs in 5′-UTR of LMO1

All RNA samples were DNase-treated (Qiagen) prior to Superscript III RT-PCR (Invitrogen). The SNP named rs2071485 C/T in the *LMO1* 5’-UTR region was analysed by PCR of Jurkat genomic DNA and paired Jurkat cDNA samples, and sequencing with the following primer pairs: (1) for Jurkat genomic DNA: 5′-TAGCGGGCTCTAATTACCCG-3′ and 5′-CGTCTCCACTCCCCATTAACC-3′ (2) for Jurkat cDNA: 5′-GCCACGAGATTCCCCCATCT-3′ and 5′-CGGTCCTTGATCTTGCGGTT-3′. PCR products were purified using QIAquick PCR purification kit (Qiagen) and sent for Sanger sequencing in both forward and reverse orientation (Genewiz, Inc.).

### Luciferase reporter assay

A 585-bp genomic region of the *LMO1* enhancer mutation site was cloned into pGL3-promoter vector (E176A, Promega), encoding a minimal SV40 promoter upstream of Firefly luciferase (pGL3-Luc). For reporter assays, additional information can be found in Supplementary Materials and Methods.

### Lentiviral shRNA induced MYB knockdown experiments

shRNA sequences were cloned into the lentiviral vector pLKO.1-puro. The target sequences are 5′-ACAACAGCCACAACGTCTATA-3′ (GFP shRNA) and 5′-CCAGATTGTAAATGCTCATTT-3′ (MYB shRNA).^12^ Additional information for lentiviral particles preparation and infection of T-ALL cells can be found in Supplementary Materials and Methods.

### Processing and analysis of chromatin immunoprecipitation coupled with massively parallel DNA sequencing (ChIP-seq)

ChIP coupled with massively parallel DNA sequencing (ChIP-seq) was performed and analysed as previously described.^5, 12^ For additional information, see Supplementary Materials and Methods.

### Analysis of transcription factor binding motifs

Wild type and mutant *LMO1* enhancer sequences were analysed with UniPROBE as previously published.^5, 21, 22^ Locations of DNA sequence motifs preferred by important T-ALL regulators were identified in the *LMO1* enhancer by using FIMO with motif libraries from Transfac and HOCOMOCO.^23^

### ChIA-PET experiments and the data analysis in Jurkat cells

The ChIA-PET data were obtained from a previous study (PMID:26940867, GEO ID: GSE68977).^24^ The data were processed using the Dowen pipeline as described in PMID: 26940867 with minor modifications.^24^

### Statistical analysis

Statistical significance was assessed with Student’s *t*-test (two-tailed).

## Results

### Aberrant enhancer activity associated with high-level expression of LMO1 in human T-ALL cells

To identify mutations in the T-ALL genome that might significantly alter oncogene expression, we focused our search on aberrant, sample-specific enhancers in 10 different human T-ALL cell lines as identified by H3K27ac ChIP-seq. By focusing on the small fraction of the genome enriched in H3K27ac, we eliminated much of the vast human noncoding sequence from consideration. We also focused on enhancers that were active in human T-ALL cell lines, but not evident in normal thymocytes or CD34^+^ hematopoietic stem and progenitor cells (HSPCs).

**Figure 1:**
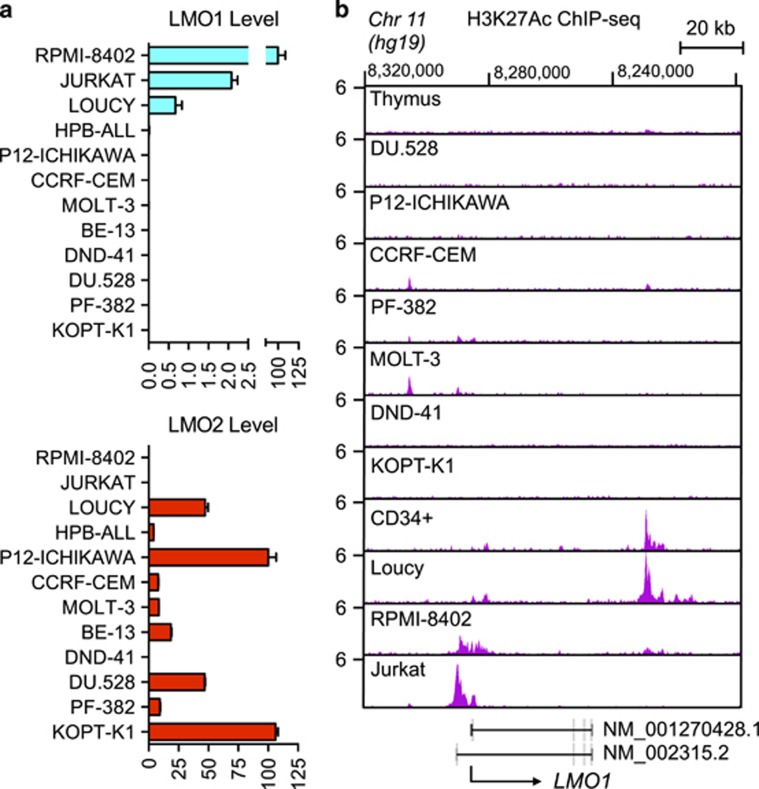
Aberrant enhancer upstream of the *LMO1* gene in RPMI-8402 and Jurkat cells. (**a**) mRNA expression of *LMO1* (upper panel) and *LMO2* (lower panel) determined by quantitative polymerase chain reaction (PCR) and normalized to human 18S ribosomal RNA in 12 human T-ALL cell lines. (**b**) Normalized ChIP-seq tracks of H3K27ac at the *LMO1* locus in human fetal thymic tissue, human purified normal hematopoietic stem cell sample (CD34^+^) that expresses *LMO1* mRNA, and 10 human T-ALL cell lines. The black arrow beneath the chart indicates the direction of *LMO1* transcription. ChIP-seq read densities (*y* axis) are normalized to reads per million reads sequenced in each sample.

Human T-ALL cell lines generally express high levels of either *LMO1* or its close relative *LMO2*, consistent with the functional redundancy of these two critical oncogenes in T-ALL transformation (Figure 1a). The three T-ALL cell lines that overexpress *LMO1* (RPMI-8402, Jurkat and Loucy) also contain active enhancer regions adjacent to the gene based on the H3K27ac ChIP-seq results shown in Figure 1b. The previously identified normal hematopoietic cell enhancer downstream of the *LMO1* gene is evident in normal CD34^+^ HSPCs^20^ and in the Loucy cell line (Figure 1b), which has an early T-cell precursor (ETP ALL) phenotype.^25^ The aberrant active enhancer we identified was present upstream of the *LMO1* gene in RPMI-8402 and Jurkat T-ALL cell lines, but not in normal CD34^+^ HSPCs or normal thymus (Figure 1b). RPMI-8402 cells contain the t(11;14)(p15;q11) *LMO1-TCRD* chromosomal translocation, which juxtaposes *LMO1* to gene regulatory elements within the T-cell receptor α/δ locus, leading to ectopic overexpression of *LMO1*.^7^ Jurkat cells, by contrast, lack a chromosomal translocation to account for aberrant *LMO1* enhancer activity and high levels of expression of the oncogene, suggesting unknown *cis*-acting genomic lesions affecting *LMO1* regulatory sequences that create the aberrant transcriptional enhancer in Jurkat cells.

### Identification of a recurrent, somatic, heterozygous C-to-T single nucleotide mutation in T-ALL

**Figure 2:**
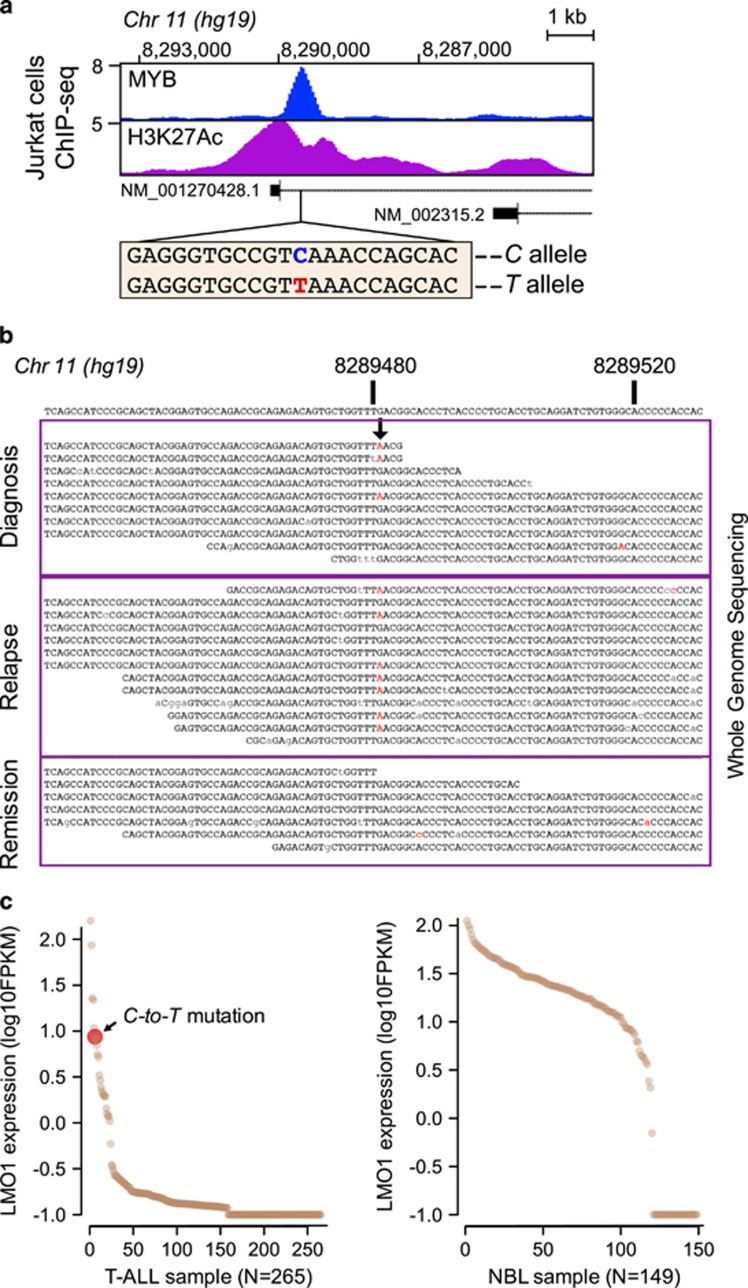
A C-to-T single nucleotide mutation is somatically acquired in human T-ALL. (**a**) Identification of a heterozygous C-to-T single nucleotide substitution that aligns precisely with the MYB ChIP-seq peak in Jurkat cells, designated *C* allele and *T* allele, respectively. (**b**) Whole genome sequencing (WGS) reads from diagnosis (top), relapse (middle) and remission (bottom) DNA of a T-ALL patient with the C-to-T mutation (shown as G-to-A and pointed by the arrow). Mismatch to the reference genome, which represents the mutant allele, is labelled in red. Lower-case letters are used to present residues with low sequence quality (quality score <20). (**c**) Scatterplot of *LMO1* expression in pediatric T-ALL (left panel) and neuroblastoma (right panel) by RNA-seq data generated by the TARGET project. The T-ALL sample with the C-to-T mutation is marked in red and by an arrow.

The aberrant enhancer in Jurkat cells is located ~4 kb upstream of the proximal transcription start site of *LMO1*, which is used exclusively by these cells based on polyA RNA-seq analysis (Figure 1b and Supplementary Figure S1). ChIP-seq results showed precise alignment of MYB binding and H3K27ac accumulation at the site of the aberrant *LMO1* enhancer in Jurkat cells (Figure 2a), which prompted us to perform Sanger sequence analysis of the genomic DNA region encompassing the MYB binding peak by ChIP-seq. We identified a heterozygous C-to-T single nucleotide mutation that aligned precisely with the MYB ChIP-seq peak in Jurkat cells (Figure 2a and Supplementary Figure S2). None of the remaining 19 human T-ALL cell lines had any detectable genomic sequence abnormalities in this region (Supplementary Table S1).

Interestingly, we found that the mutation we identified, TCA to TTA, conforms to an APOBEC mutational signature, TCN to TTN, which has been widely identified across the genome in a variety of human cancer types as 'signature 2'.^26, 27^ APOBEC3 was highly expressed during thymocyte development in the mouse (Supplementary Figure S3A).^28^ There are 11 distinct human APOBEC family members.^29^ Human T-ALL cell lines expressed varying levels of APOBEC3B, 3C and 3G (Supplementary Figure S3B). Consistently, RNA-seq data of 265 primary T-ALLs (Figure 2c) revealed high levels of expression of APOBEC3C, 3D, 3 F and 3G in all cases and expression of APOBEC3A, 3B and 3H in a subset of cases (Supplementary Figure S3C). These results indicate ample opportunity for APOBEC mutations to occur during aberrant thymocyte development leading to T-ALL.

Sequencing of 187 paediatric primary T-ALL samples collected at diagnosis identified 4 patients (2.14%) who harbour the same heterozygous mutation. In three of these patients who had matched diagnosis and remission samples, the mutations were somatically acquired in the malignant T-ALL clone (Figure 2b and Supplementary Figure S4). The somatic mutation was also identified in the relapse sample available for one of these patients (Figure 2b). The somatic origin of the mutant allele was further confirmed by its absence in the germline WGS data generated from 2925 paediatric cancer patients. Two of the four primary T-ALLs with *LMO1* enhancer mutation harboured SIL-TAL1 deletion and one carried TCR-TAL2 translocation (Supplementary Table S2), indicating that they belong to the TAL1/TAL2^+^ molecular subtype of T-ALL, consistent with the notion that TAL and LMO proteins act as a complex and function cooperatively in T-ALL transformation.^30, 31^ None of these four cases had characteristics of early T-cell precursor (ETP) ALL, in that the *TCR*γ chain was rearranged in the three cases that were tested and the immunophenoytpe of each case did not meet the criteria for ETP ALL.^32, 33^

Analysis of the available RNA-seq data showed that the T-ALL tumour with the *LMO1* enhancer mutation had the sixth highest expression level of *LMO1* relative to the 264 T-ALLs analysed in the NCI TARGET (Figure 2c), consistent with the cancer cell line data (Figure 1a). This finding emphasizes the importance of the matched gene expression data in interpreting the consequences of noncoding mutations and shows that the C-to-T mutation leads to high expression levels of *LMO1* in patient samples as well as in Jurkat cells. We have previously shown that a common single nucleotide polymorphism in the first intron of the *LMO1* gene is highly associated with *LMO1* expression in neuroblastoma cells.^34^ RNA-seq analysis showed that ~4% of T-ALL tumours express high levels of *LMO1*, whereas 79% of neuroblastoma tumours express high levels of this gene (Figure 2c). Analysis of the sequence of this region in 214 diagnostic samples and 21 relapse samples from neuroblastoma patients identified only the reference *C* allele at this position, with no evidence for the C-to-T mutation.

### The C-to-T mutation introduces a MYB binding motif and drives LMO1 overexpression

**Figure 3:**
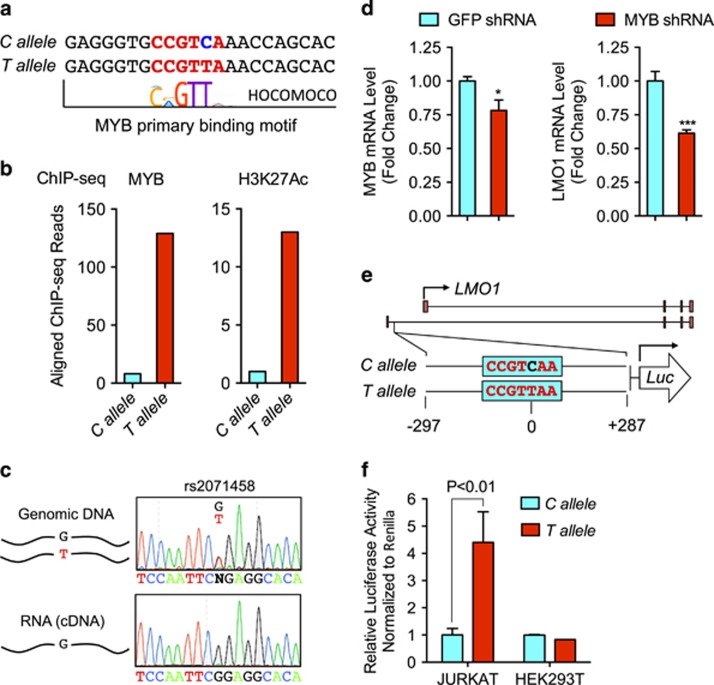
The C-to-T single nucleotide mutation creates a MYB transcription factor-binding motif and activates *LMO1* transcription through recruitment of MYB. (**a**) C-to-T substitution introduces a *de novo* MYB transcription factor-binding motif. (**b**) ChIP-Seq reads for H3K27ac and MYB preferentially align with reference sequences containing the *T* mutation. Counts of reads aligning with fragments containing *T* (*T* allele, red) and fragments containing *C* (*C* allele, blue) are displayed as bar plots. (**c**) Sanger sequencing chromatograms of genomic DNA and cDNA show that the *LMO1* gene is expressed from one allele in Jurkat cells. (top) The SNP rs2071458 shown by Sanger sequencing of genomic DNA in coding region of *LMO1* gene; (bottom) Sanger sequencing of Jurkat cDNA. (**d**) mRNA levels of *MYB* and *LMO1* determined by quantitative real-time PCR in Jurkat cells with or without lentiviral shRNA-induced MYB knockdown. *P<0.05; ***P<0.001 by two-sample, two-tailed *t* test. (**e**) A 585-bp genomic DNA fragment from either the *C* allele or *T* allele was cloned upstream of luciferase. (**f**) The luciferase constructs from (**e**) were delivered into Jurkat and HEK293T cells. In 36 h, the firefly luciferase activity was measured, normalized to renilla luciferase and expressed as a ratio relative to activity of the reference C allele enhancer construct. **P<0.01 by two-sample, two-tailed *t*-test.

Analysis of the genomic sequences of both *C* and *T* alleles, using UniPROBE and HOCOMOCO databases,^21, 23^ identified a *de novo*-binding motif for the MYB transcription factor (Figure 3a and Supplementary Table S3), while analysis of the MYB and H3K27ac ChIP-seq DNA sequence reads aligned with this site demonstrated that MYB and H3K27ac were bound almost exclusively by the *T* allele (Figure 3b). Genomic and cDNA sequencing of the 5’UTR of the *LMO1* gene demonstrated monoallelic expression of *LMO1* in Jurkat cells (Figure 3c). Importantly, analysis of the whole-genome sequencing and RNA-seq data of the primary T-ALL sample with C-to-T mutation also showed monoallelic expression of *LMO1* (Supplementary Figure S5). Knockdown of *MYB* expression using lentivirus-transduced shRNA decreased the expression of *LMO1* significantly in Jurkat cells (Figure 3d), indicating that MYB binding to the somatically acquired heterozygous MYB binding motif leads to enhanced expression of *LMO1* in T-ALL from the same allele.

To ascertain whether this single base-pair substitution can activate *LMO1* gene expression, we cloned a 585-bp genomic DNA fragment from either the *C* allele or *T* allele upstream of luciferase and tested the enhancer activity of this fragment in a reporter assay (Figure 3e). When introduced into Jurkat cells, the construct containing the *T* allele exhibited robust reporter activity, which was four-fold greater than that of the fragment containing the *C* allele (Figure 3f). However, the *T* allele reporter showed no increased activity over that of the *C* allele in human HEK-293 T cells (Figure 3f), suggesting that MYB and other members of the TAL1 complex expressed by Jurkat cells are required for the activation of gene expression from this site.^5, 12^ Taken together, we have shown that the somatically acquired C-to-T mutation that creates a MYB binding motif ~4 kb upstream of the proximal transcription start site of *LMO1* in T-ALL can generate an active transcriptional enhancer that drives monoallelic overexpression of the *LMO1* oncogene.

### MYB binding leads to a large aberrant enhancer upstream of the LMO1 start site

**Figure 4:**
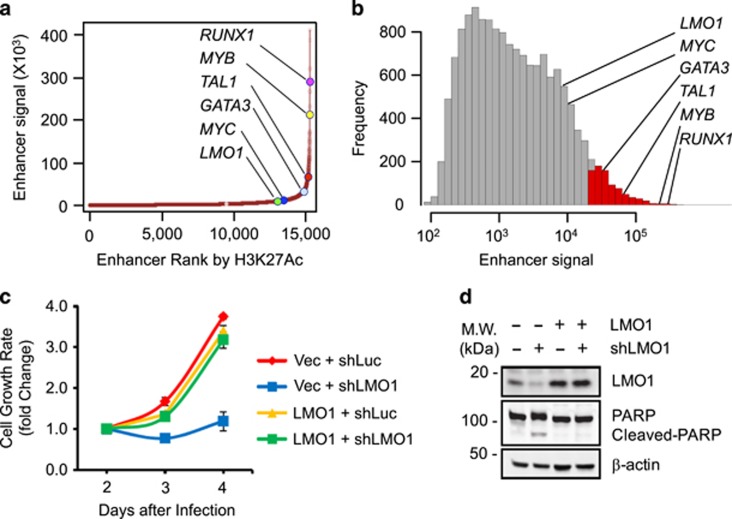
Analysis of the *LMO1* aberrant active enhancer and the requirement of *LMO1* expression for cell survival in Jurkat cells. (**a**) Distribution of H3K27ac ChIP-seq signal at enhancers in Jurkat cells. Enhancer regions are plotted in increasing order based on their input-normalized H3K27ac ChIP-seq signal. (**b**) Frequency distribution of H3K27ac ChIP-seq enhancer signal (log_10_) in Jurkat cells. Enhancers qualifying as 'super-enhancers' are shown as red bars. (**c**) Knockdown of *LMO1* by lentivirus-transduced shRNA decreased cell viability in Jurkat cells. (**d**) Western blot results show the protein levels of LMO1 and PARP cleavage in control and LMO1 overexpressing Jurkat cells after LMO1 knockdown.

Our discovery of an aberrant oncogenic enhancer upstream of the *LMO1* gene differs in important ways from the super-enhancer we identified upstream of the *TAL1* gene.^5^ Although enhancer mutations in both cases create a *de novo* MYB binding site, which in turn initiates a large aberrant enhancer that drives expression of a T-ALL oncogene, the mutations upstream of *TAL1* consist of 2- to 18-bp insertions, while in the case of *LMO1*, the causal mutation is a C-to-T single nucleotide transition. Another difference is that, although the *LMO1* enhancer is quite large, it does not meet the strict definition of a 'super-enhancer', as shown in Jurkat cells by both an enhancer rank chart (Figure 4a) and a frequency distribution of the enhancer signal (log_10_; Figure 4b). As we and others have shown for *TAL1*,^12, 35^ knockdown of *LMO1* transcripts by lentivirus-transduced shRNA in Jurkat cells demonstrates that high levels of LMO1 expression are required for cell survival (Figures 4c and d), emphasizing that aberrant enhancer elements can alter gene expression sufficiently to qualify as oncogenic drivers in T-ALL, despite their lack of full super-enhancer status.

### Binding of other members of TAL1 complex to the LMO1 enhancer in Jurkat cells

**Figure 5:**
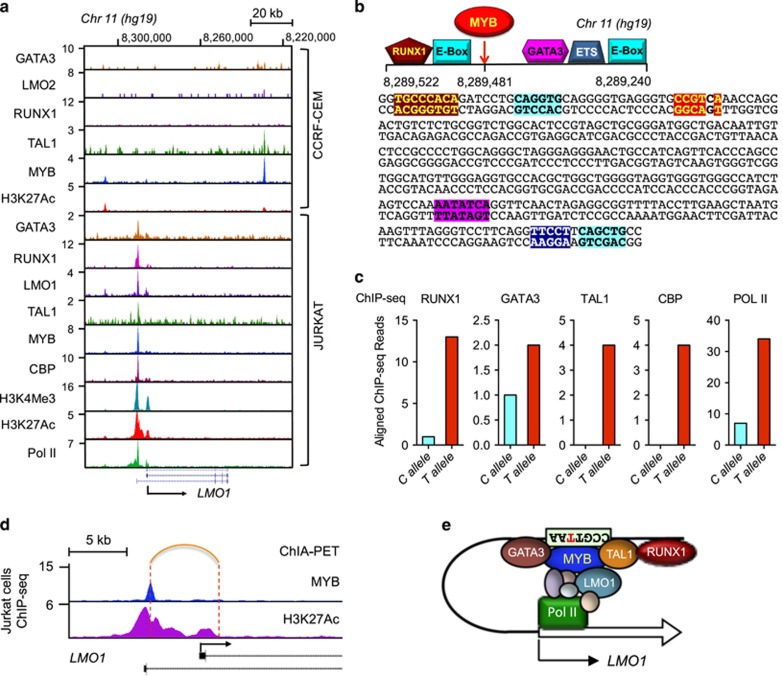
MYB binding initiates binding of other members of TAL1 complex to the aberrant *LMO1* enhancer. (**a**) ChIP-seq tracks at the *LMO1* locus for GATA3, LMO2, LMO1, RUNX1, TAL1, MYB, CBP, H3K4me3, H3K27ac, and RNA polymerase II (Pol II) in CCRF-CEM and Jurkat cells. (**b**) (upper) Schematic depiction of the region flanking the C-to-T mutation site (*Chr 11: 8,289,481 (hg19)*), showing binding sites for members of the TAL1 complex. (lower). Sense and antisense strands of DNA of the enhancer regions in the wild-type reference genome highlighting sequence motifs for TAL1 complex members. (**c**) ChIP-seq read counts for sequences immunoprecipitated by antibodies to RUNX1, GATA3, TAL1, CBP and Pol II, aligned with either the reference *C* allele (blue) or the mutant *T* allele (red). (**d**) ChIA-PET result showing that the C-to-T mutation site interacts with DNA 1.7 kb downstream of the proximal transcription start site of *LMO1*. (**e**) Model of transcription factor binding in the aberrant MYB-initiated enhancer complex that interacts with the promoter region of *LMO1* in Jurkat cells.

By ChIP-seq analysis in Jurkat cells, we found that MYB binding to the C-to-T mutation site in Jurkat cells is precisely aligned with the binding of other core components of the TAL1 complex, including GATA3, RUNX1 and TAL1, as well as LMO1 itself (Figure 5a).^5, 12^ These transcription factors do not bind near this site in the CCRF-CEM cell line, which lacks the C-to-T substitution, underscoring the biological importance of the C-to-T mutation that mediates MYB binding, and is required to initiate an active enhancer in this region (Figure 5a). Careful analysis of the sequence of the reference genome near the mutation site identified the preferred binding motifs for RUNX1, GATA3 and ETS1, as well as E-box motifs characteristic of binding by TAL1-E2A heterodimers (Figure 5b). Analysis of the DNA sequences immunoprecipitated in the ChIP-seq experiments showed that RUNX1, GATA3, TAL1, CBP and Pol II each binds preferentially to the allele with the T base-pair mutation in Jurkat cells, indicating that the enhancer activity occurs only after MYB binding, subsequent to the acquisition of this single base-pair substitution (Figure 5c). We also used chromatin interaction analysis with paired-end tag sequencing (ChIA-PET)^24^ in Jurkat cells to demonstrate that the aberrant *LMO1* enhancer interacts with a region 1.7 kb downstream of the proximal *LMO1* transcription start site used by Jurkat cells (Figure 5d and Supplementary Figure S1), indicating that the active enhancer mediated by the acquired MYB binding motif loops to the *LMO1* gene promoter region to regulate the transcription of *LMO1* (Figure 5e).

## Discussion

A major question related to noncoding mutations that give rise to oncogenic enhancer elements is whether these enhancers are tissue-type specific or whether the same elements can function in different types of tumours. We recently reported that genetic predisposition to neuroblastoma is mediated by an inherited G-T polymorphism that controls a super-enhancer within the first intron of *LMO1* in these cells,^34^ promoting us to compare regulation of the *LMO1* oncogene in neuroblastoma with that in T-ALL. We did not find the C-to-T mutation in 214 diagnostic samples and 21 relapse samples from neuroblastoma patients, indicating the mutation-induced enhancer that activates *LMO1* expression in T-ALL does not arise during clonal evolution in neuroblastoma, emphasizing the context-dependence of this mode of transcriptional regulation. Similarly, even though GATA3 is highly expressed in T-ALL cells,^12^ the GATA3 binding site in the first intron of *LMO1*, which mediates super-enhancer formation in neuroblastoma,^34^ is not bound by GATA3 in T-ALL cells (compare Figure 5a with 3a in Oldridge *et al.*^34^). These findings illustrate the complex nature of lineage-specific enhancer formation in tumorigenesis as well as development, and underscore the need for further research into the lineage-restricted factors required for enhancer formation.

Different mutational processes leave characteristic signatures in cancer genomes, which implicate the mechanisms underlying the somatic mutations that arise during the evolution of human tumours.^26, 36^ The somatic insertions upstream of *TAL1* oncogene that we recently identified in T-ALL are likely acquired due to the expression of RAG1 and RAG2 in early lymphoid cells.^5, 6^ The C-to-T mutation described here occurs in the context of a TCA motif, corresponding to one of the APOBEC-induced mutational signatures.^26, 29, 36^ We recognize that C-to-T transitions can be caused by mechanisms other than the APOBEC deaminases;^36^ however, somatic mutations with the distinctive APOBEC signature mutations have been widely identified across the genome in a variety of human cancer types, including breast cancer, lung cancer and acute myeloid leukaemia, as well as acute lymphoblastic leukaemia.^26, 27, 29, 36, 37, 38^ In a recent study^39^ of 560 breast cancers and matched non-neoplastic tissue, >70% of the tumours harboured from 10 to 90 000 mutations whose signatures reflected aberrant APOBEC DNA-editing activity, and most of these were located within the noncoding regions of the genome. Cancer risk variants that affect the noncoding genome pose difficult challenges such as discriminating between 'driver' and 'passenger' mutations. Our studies provide a clear example of how noncoding single base pair substitutions can function as oncogenic drivers by introducing a single transcription factor-binding site at a strategic location in the patient’s genome. Aberrant binding of the specific transcription factor, in this case MYB, can then initiate the formation of an oncogene-specific enhancer complex that drives high levels of oncogene expression.

Despite the rapid and revolutionary advancement of genomic sequencing technologies over the past decade, efforts to identify 'driver mutations' in the noncoding genome of cancer cells have been challenging because the noncoding genome, exclusive of transposons, repeated sequence elements and heterochromatin, is at least 30 times larger and more complex than the coding genome.^40^ To identify and distinguish 'driver mutations' from 'passenger mutations' in noncoding genome of cancer, it will be important to perform both H3K27ac ChIP-seq and RNA-seq, in addition to whole-genome sequencing, for each tumour sample. The H3K27ac ChIP-seq results allow one to focus on mutations that occur within active enhancers, which greatly reduce the number of mutations that could act in this manner. Since mutations that upregulate oncogene expression are often heterozygous, the sequence of the DNA fragments precipitated in the ChIP-seq procedure should preferentially include the mutated compared with the reference sequence. RNA-seq should then indicate high levels of expression of the target gene, and the presence of SNPs within the expressed sequences should reveal preferential expression of one of the two alleles. As illustrated in our study, this experimental approach should readily identify *bona fide* driver mutations that act by initiating aberrant enhancers within the noncoding genome of human cancers. Regulatory mutations of this type provide clear evidence for selection during clonal evolution, and thus both the oncoprotein that is upregulated and the aberrantly activated transcriptional machinery provide attractive targets for therapeutic inhibition.^41, 42^
