# Supplementary material for: APOBEC signature mutation generates an oncogenic enhancer that drives LMO1 expression in T-ALL
Source: Leukemia. 2017 Mar 28;31(10):2057–64. doi: 10.1038/leu.2017.75 (PMC5629363; doi:10.1038/leu.2017.75)
Supplement: Supplementary Information [file leu201775x1.docx]

**SUPPLEMTARY MATERIALS AND METHODS**

***T-ALL patient samples and sequencing analysis (UKALL trial from United Kingdom)***

Diagnostic genomic DNA was available from 49 adult (>18yrs old) and 130 paediatric patients (<=18 yrs old) with T-ALL. Genomic DNA from the time of relapse with T-ALL was also available from a further 12 adult and 8 paediatric patients. The majority of the paediatric patients with diagnostic samples were enrolled on the UKALL2003 trial (1) and registration of the trial can be found at http://www.controlled-trials.com under ISRCTN number 07355119. Ethical approval for the trial was obtained from the Scottish Multi-Centre Research Ethics Committee. The majority of the adult patients with diagnostic samples were enrolled on the UKALL14 trial, ISRCTN registration number 66541317 with ethical approval obtained from West London REC 2. The remaining diagnostic and relapse samples were obtained from patients with T-ALL referred to University College Hospital. All samples were collected from patients with informed consent according to the Declaration of Helsinki.

Mutation screening of the *LMO1* intron 1 enhancer region was performed using PCR of genomic DNA and hetero-duplex analysis using the WAVE DNA Fragment Analysis System (Transgenomic, Glasgow, UK). The primers were designed to amplify a 304 bp product in the *LMO1* intron 1 enhancer region that includes the guanine nucleotide residue at position chr11:8289481 (GRCh37/hg19), found to be mutated in the Jurkat cell line (Forward 5’-GATGGCAGTTCCCTCCCTAG-3’ and Reverse 5’-CTTTCTCACGGACTCTTGCC-3’). 20 μl PCR reactions were done using 10 μl Phusion High-Fidelity PCR Master Mix with HF Buffer (New England Biolabs, Hitchin, UK), 0.5 µM of each primer, 2.5% of DMSO, 6.5 μl of PCR-grade water, and 1 μl of template DNA. The cycling parameters were: 98 ^0^C for 60 s, followed by a 35-cycle amplification with denaturing at 98 ^0^C for 20 s, annealing at 60 ^0^C for 20 s and extension for 72 ^0^c for 30 s, followed by further extension at 72 ^0^C for 5 mins. All products were analysed by electrophoresis on 2% agarose gels. All products from the PCR reactions were then denatured, and re-annealed slowly to allow for heteroduplex formation and analysed by High Pressure Liquid Chromatography on the WAVE DNA Fragment Analysis system (Transgenomic, Glasgow, UK). Optimal denaturing and annealing temperatures were calculated using the Transgenomic Navigator Software and each sample was analysed at 2 melting temperatures, 64.8 ^0^C and 65.3 ^0^C. Any sample with an abnormal chromatogram was Sanger sequenced using the forward and reverse PCR primers.

***T-ALL Patient Samples and sequencing analysis (Shanghai Children’s Medical Center and St Jude Children’s Research Hospital)***

The *LMO1* enhancer mutation identified in this study was screened in matched tumour-normal samples of 57 T-ALL patients from Shanghai Children’s Medical Center (SCMC) and St Jude Children’s Research Hospital (SJCRH). These samples included 19 diagnosis-relapse-remission triads and 38 diagnosis-remission pairs, all analysed by whole-genome sequencing (WGS) at an average of 30X coverage. The WGS paired-end reads were aligned to the reference human genome assembly GRCh37-lite using BWA (2), and variants were detected as previously described (3).

Transcriptome sequencing (RNA-seq) data from cases of T-ALL with the somatic *LMO1* mutation was used for analysing aberrant expression levels and the allelic imbalance signature of *LMO1*. The expression of RNA-seq was measured as the FPKM value based on transcript models in GENCODE v19 using HTseq-count (4); and was compared with values derived from RNA-seq of 264 T-ALL samples analysed in the TARGET (Therapeutically Applicable Research to Generate Effective Treatments, https://ocg.cancer.gov/programs/target) project of NCI.

To compared WGS against RNA-seq to assess the allelic imbalance of *LMO1* expression in the T-ALL with the enhancer mutation, a total of 18 heterozygous germline SNPs located within the 44 kb *LMO1* genomic region (chr11:8,245,851-8,290,182) with a variant allele frequency (VAF) ranging from 0.3 to 0.7 by tumour WGS (>10X read-coverage in both WGS and RNA-seq), were selected.

***Neuroblastoma (NBL) Patient Samples and sequencing analysis***

A total number of 235 matched tumour-normal NBL samples, from SJCRH(5) and the Therapeutically Applicable Research to Generate Effective Treatments (TARGET) study (https://ocg.cancer.gov/programs/target), were included for screening *LMO1* C-to-T enhancer mutation identified in this study. The patient samples included 214 diagnosis samples and 21 relapse samples. All samples were analysed by WGS (using Illumina platform for SJCRH cohort (N=66) and Complete Genomics platform for TARGET cohort (N=169)). 149 RNA-seq data of diagnosis samples from TARGET study were included for *LMO1* expression analysis, as described in T-ALL section. All WGS and RNA-seq data from TARGET study were downloaded from dbGaP under accession number phs000467 (PMID: 23334666).

***Luciferase reporter assay***

A 585-bp genomic region of the *LMO1* enhancer mutation site was amplified from Jurkat genomic DNA by 35 cycles of PCR using Phusion reagents (Finnzymes) and primers 5’-GGCGAGCTCAGGCTCAGCGCCCGGGCTTC and 5’-GCTCTCGAGGAAAAGGACTACAAGACTAC. PCR products were digested with SacI and XhoI (New England Biolabs), and cloned into the respective sites of the pGL3-promoter vector (E176A, Promega), encoding a minimal SV40 promoter upstream of Firefly luciferase (pGL3-Luc). For reporter assays, 1x10^6^ Jurkat cells were suspended in 100 μl of Nucleofector Solution (Mirus) with the addition of 1 μg of pGL3-Luc and 1μg of renilla plasmid (pTK), electroporated on program D-23 (Amaxa), resuspended in 500μl of RPMI/10%FCS and incubated at 37°C/5% CO_2_ for 48 hr. Luciferase activity was measured using the Dual-Glo Luciferase Assay system (Promega) according to the manufacturer’s instructions. Experiments were performed in triplicate. Firefly luciferase activity was normalized to renilla luciferase and measurements were expressed as a ratio relative to activity of the wild-type *LMO1* enhancer construct.

***Lentiviral shRNA induced MYB knockdown experiments***

shRNA sequences were cloned into the lentiviral vector pLKO.1-puro. The target sequences are ACAACAGCCACAACGTCTATA (GFP shRNA) and CCAGATTGTAAATGCTCATTT (MYB shRNA) (6). Each construct was cotransfected into 293T cells with an envelope plasmid pMD2.G and packaging plasmids pMDLg/pRRE and pRSV-REV, using FuGENE 6 reagent (Roche). Supernatants containing the lentivirus were collected and filtered with a 0.45 µm filter (Millipore). Jurkat cells were infected with lentivirus in the presence of polybrene (8 ug/ml) and HEPES (10 mM) by centrifugation at 2,500 rpm for 1.5 hr at 30 °C, and the infected cells were selected by puromycin for at least 36 hr. Total RNA was extracted using a NucleoSpin RNA kit (Macherey-Nagel) and was reverse transcribed with a QuantiTect reverse transcription kit (Qiagen). Quantitative PCR analysis was performed with an Applied Biosystems 7300 Real Time PCR System (Applied Biosystems). The qPCR primers were used as described previously (6).

***Chromatin immunoprecipitation coupled with massively parallel DNA sequencing (ChIP-seq)***

ChIP coupled with massively parallel DNA sequencing (ChIP-seq) was performed as previously described (6, 7). The following antibodies were used for ChIP: anti-H3K27ac (Abcam, ab4729), anti-H3K4me3 (Abcam, ab8580), anti-Pol II (Abcam, ab26721), anti-cMYB (Abcam, ab45150), anti-TAL1 (Santa Cruz, sc-12984), anti-GATA3 (Santa Cruz, sc-22206), anti-RUNX1 (Santa Cruz, sc-8563), anti-CBP (Abcam, ab14984), anti-LMO1 (Santa Cruz, sc-10494), and anti-LMO2 (R&D, AF2726). For each ChIP, 10 μg of antibody was added to 3 ml of sonicated nuclear extract. Illumina sequencing, library construction and ChIP-seq analysis methods were described in earlier publications (6, 7).

***ChIP-seq processing and analysis***

ChIP-seq reads were aligned to the hg19 revision of the human reference genome using bowtie (8) with parameters –k 2 –m 2 –best –sam and –l set to the read length. Wiggle files for display were created with MACS (9) with parameters –space=50 –nomodel –shiftsize=200, and were subsequently normalized by dividing read counts per bin by the millions of mapped reads. Enhancers were defined as having enrichment in H3K27ac. Super-enhancers were defined as previously reported (7). Briefly, two sets of H3K27ac-enriched regions were identified with MACS with input control and parameters –p 1e-9 –keep-dup=auto and –p 1e-9 –keep-dup=all. These were collapsed into one peak set and used as input for ROSE (https://bitbucket.org/young_computation/rose/) with parameters –s 12500 –t 2000 –g hg19. Stitched enhancers (typical enhancers and super-enhancers) were assigned to the single most proximal active RefSeq gene whose transcription start site was nearest the center of the enhancer. Active genes were in the top two-thirds of genes ranked by promoter (TSS +/- 500) H3K27ac density as calculated with bamToGFF (https://github.com/BradnerLab/pipeline) with parameters –e 200 –m1 –r –d. The accession code for all the ChIP-seq data in this study are as following:

Loucy H3K27ac: GSM2037788; DU.528 H3K27ac: GSM2037785; RPMI-8402 H3K27ac: GSM1442003; CCRF-CEM H3K27ac: GSM2037781; PF-382 H3K27ac: GSM2037796; Jurkat H3K27ac: GSM1296384; P12-ICHIKAWA H3K27ac: GSM2037794; DND41 H3K27ac: SRR568222; KOPT-K1 H3K27ac: GSM2318734; MOLT3 H3K27ac: GSM1519644; Thymus H3K27ac: GSM1013125; CD34+ cells H3K27ac: GSM772870; Jurkat GATA3: GSM1975913; Jurkat TAL1: GSM722165; Jurkat RUNX1: GSM1697879; Jurkat LMO1: GSM2474553; Jurkat MYB: GSM1442006; Jurkat CBP: GSM449527; Jurkat H3K4me3: GSM1464996; Jurkat Pol II: GSM1224784; CCRF-CEM GATA3: GSM1442006; CCRF-CEM LMO2: GSM837993; CCRF-CEM RUNX1: GSM837995; CCRF-CEM TAL1: GSM837997

**REFERENCES:**

1. Vora A, Goulden N, Wade R, Mitchell C, Hancock J, Hough R, et al. Treatment reduction for children and young adults with low-risk acute lymphoblastic leukaemia defined by minimal residual disease (UKALL 2003): a randomised controlled trial. Lancet Oncol. 2013;14(3):199-209.

2. Li H, Durbin R. Fast and accurate short read alignment with Burrows-Wheeler transform. Bioinformatics. 2009;25(14):1754-60.

3. Zhang J, Ding L, Holmfeldt L, Wu G, Heatley SL, Payne-Turner D, et al. The genetic basis of early T-cell precursor acute lymphoblastic leukaemia. Nature. 2012;481(7380):157-63.

4. Anders S, Pyl PT, Huber W. HTSeq--a Python framework to work with high-throughput sequencing data. Bioinformatics. 2015;31(2):166-9.

5. Cheung NK, Zhang J, Lu C, Parker M, Bahrami A, Tickoo SK, et al. Association of age at diagnosis and genetic mutations in patients with neuroblastoma. JAMA. 2012;307(10):1062-71.

6. Sanda T, Lawton LN, Barrasa MI, Fan ZP, Kohlhammer H, Gutierrez A, et al. Core transcriptional regulatory circuit controlled by the TAL1 complex in human T cell acute lymphoblastic leukemia. Cancer Cell. 2012;22(2):209-21.

7. Mansour MR, Abraham BJ, Anders L, Berezovskaya A, Gutierrez A, Durbin AD, et al. Oncogene regulation. An oncogenic super-enhancer formed through somatic mutation of a noncoding intergenic element. Science. 2014;346(6215):1373-7.

8. Langmead B, Trapnell C, Pop M, Salzberg SL. Ultrafast and memory-efficient alignment of short DNA sequences to the human genome. Genome Biol. 2009;10(3):R25.

9. Zhang Y, Liu T, Meyer CA, Eeckhoute J, Johnson DS, Bernstein BE, et al. Model-based analysis of ChIP-Seq (MACS). Genome Biol. 2008;9(9):R137.

**SUPPLEMTARY FIGURE LEGENDS**

**Figure S1: RNA-seq result shows the exclusive usage of the proximal transcription start site of *LMO1* in Jurkat cells.**

**Figure S2: A C-to-T single nucleotide transition identified in human T-ALL Jurkat cells.** Sanger sequencing traces show the *LMO1* C-to-T heterozygous single nucleotide mutation in Jurkat cells (bottom), but not in Loucy cells (top).

**Figure S3: Expression of APOBEC family members during thymocyte development and in T-ALL.** (A) Heatmap results based on data from Gene Expression Commons showing the expression of APOBEC family members during murine thymocyte development. (B) Expression heatmap of APOBEC family members in 16 human T-ALL cell lines as determined by Affymetrix gene expression array. (C) Scatterplot of the expression of 11 APOBEC family members in paediatric T-ALL by RNA-seq data generated by the TARGET project.

**Figure S4: Somatic *LMO1* enhancer mutation detected in primary T-ALL patients.** Sequences of a region in the *LMO1* intron 1 enhancer showing a heterozygous G-to-A (C-to-T) mutation in diagnostic genomic DNA samples of 2 patients (5532 and 6217) that is not present in the remission samples of the same patients.

**Figure S5: Monoallelic expression of *LMO1* in a primary T-ALL patient harbouring the C-to-T somatic enhancer mutation (indicated by the black arrow).** Variant allele frequency (VAF) in tumour DNA and RNA of the 18 SNPs selected for assessing allelic imbalance in RNA-seq are shown at the top. All SNPs exhibit monoallelic expression of *LMO1* gene in RNA-seq. The expression level of *LMO1* is shown below as a wiggle plot.

**SUPPLEMENTARY TABLES**

Table S1: Key genomic abnormalities, inherited G/T single nucleotide polymorphism (SNP) rs2168101, and mutational status of the LMO1 enhancer in 21 T-ALL cell lines.

Table S2: Charicteristics of patients' T-ALL samples that harbours the C-to-T LMO1 enhancer mutation from UKALL trial, United Kingdom and Shanghai Children’s Medical Center (SCMC), China.

Table S3: Transcriptional factor binding sites of C and T allele as analyzed by UniPROBE. The sequences were analyzed at: http://the_brain.bwh.harvard.edu/uniprobe/
