# Supplementary material for: APOBEC signature mutation generates an oncogenic enhancer that drives LMO1 expression in T-ALL
Source: Leukemia. 2017 Mar 28;31(10):2057–64. doi: 10.1038/leu.2017.75 (PMC5629363; doi:10.1038/leu.2017.75)

Figure S1: RNA-seq result shows the exclusive usage of the proximal transcription start site of *LMO1* in Jurkat cells.

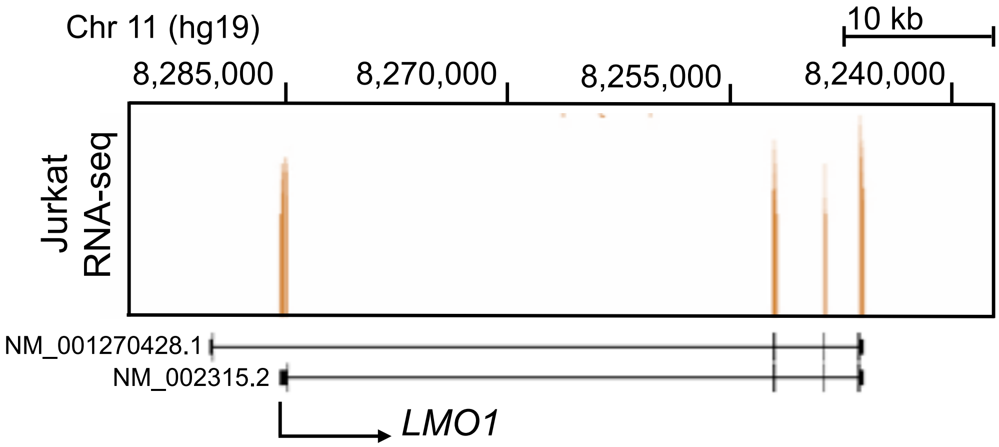

Supplement: Supplementary Figure 1 [file leu201775x2.pdf]
