# Supplementary material for: APOBEC signature mutation generates an oncogenic enhancer that drives LMO1 expression in T-ALL
Source: Leukemia. 2017 Mar 28;31(10):2057–64. doi: 10.1038/leu.2017.75 (PMC5629363; doi:10.1038/leu.2017.75)

Figure S2: A C-to-T single nucleotide transition identified in human T-ALL Jurkat cells. Sanger sequencing traces show the *LMO1* C-to-T heterozygous single nucleotide mutation in Jurkat cells (bottom), but not in Loucy cells (top).

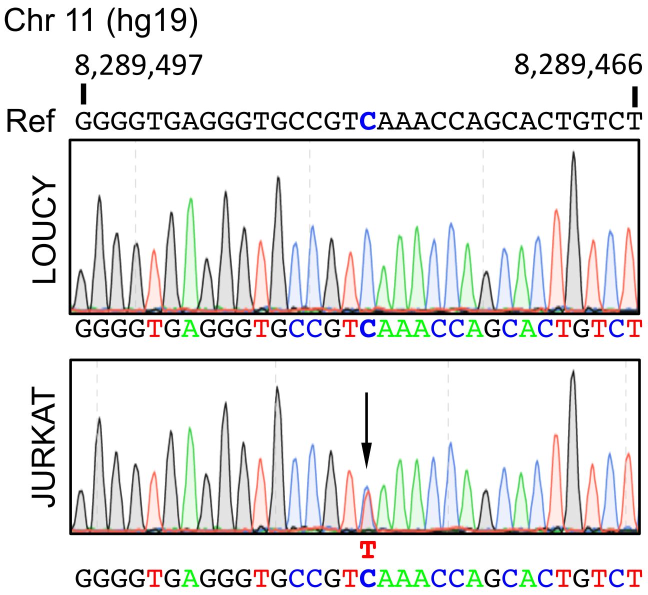

Supplement: Supplementary Figure 2 [file leu201775x3.pdf]
