# Supplementary material for: APOBEC signature mutation generates an oncogenic enhancer that drives LMO1 expression in T-ALL
Source: Leukemia. 2017 Mar 28;31(10):2057–64. doi: 10.1038/leu.2017.75 (PMC5629363; doi:10.1038/leu.2017.75)

Figure S3: Expression of APOBEC family members during thymocyte development and in T-ALL. (A) Heatmap results based on data from Gene Expression Commons showing the expression of APOBEC family members during murine thymocyte development. (B) Expression heatmap of APOBEC family members in 16 human T-ALL cell lines as determined by Affymetrix gene expression array. (C) Scatterplot of the expression of 11 APOBEC family members in paediatric T-ALL by RNA-seq data generated by the TARGET project.

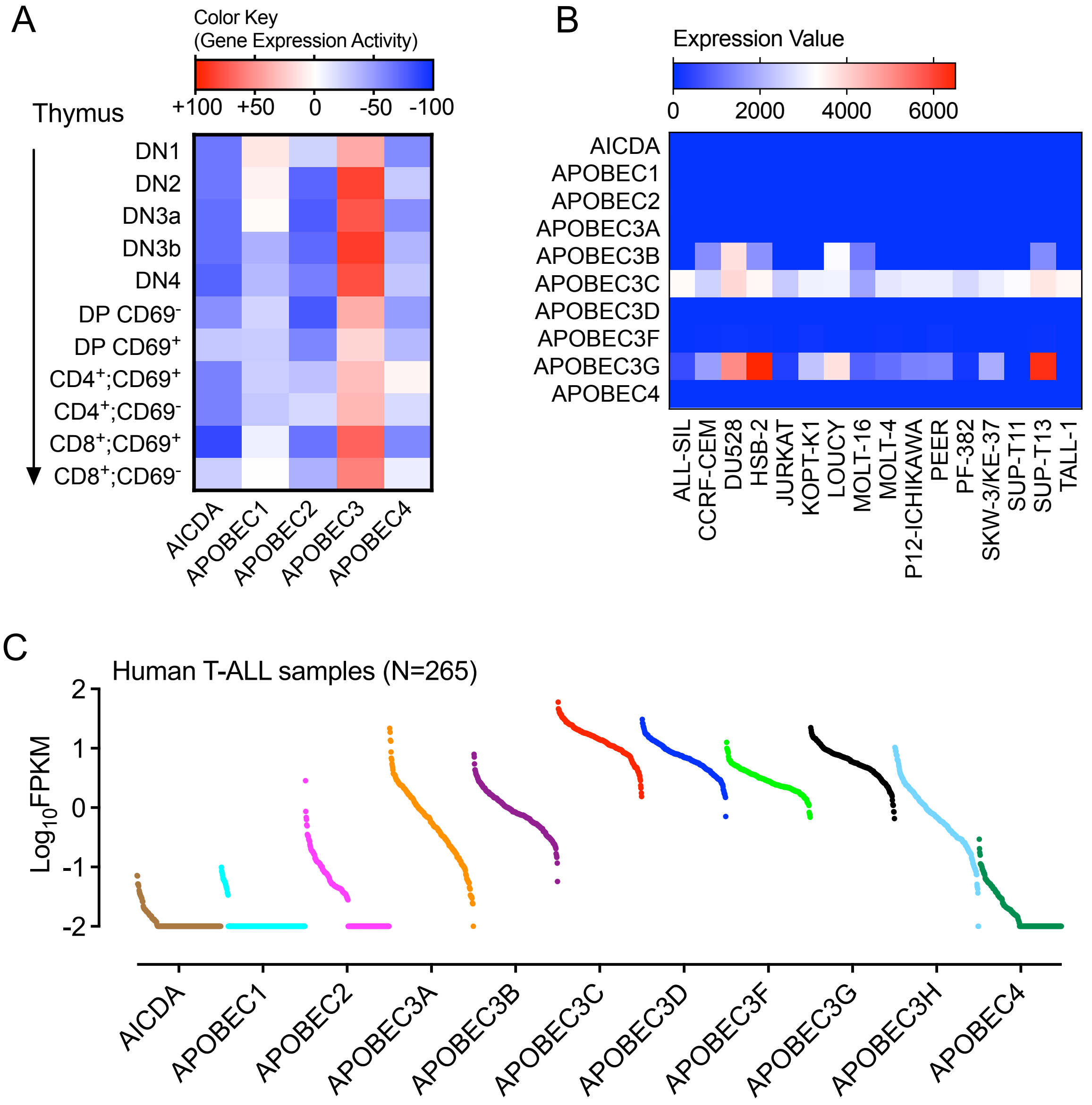

Supplement: Supplementary Figure 3 [file leu201775x4.pdf]
