# Supplementary material for: APOBEC signature mutation generates an oncogenic enhancer that drives LMO1 expression in T-ALL
Source: Leukemia. 2017 Mar 28;31(10):2057–64. doi: 10.1038/leu.2017.75 (PMC5629363; doi:10.1038/leu.2017.75)

**Figure S4: Somatic *LMO1* enhancer mutation detected in primary T-ALL patients.** Sequences of a region in the *LMO1* intron 1 enhancer showing a heterozygous G-to-A (C-to-T) mutation in diagnostic genomic DNA samples of 2 patients (5532 and 6217) that is not present in the remission samples of the same patients.

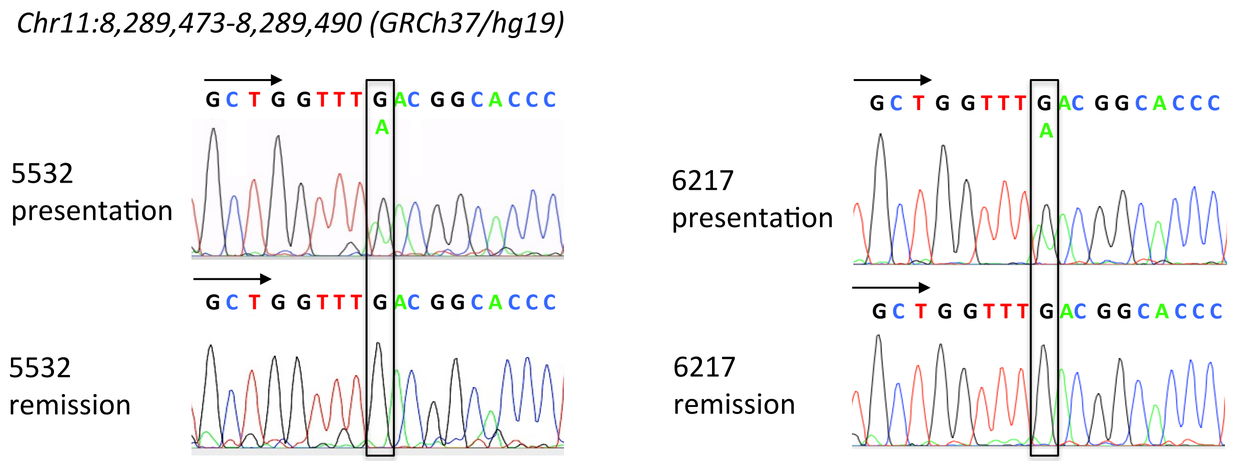

Supplement: Supplementary Figure 4 [file leu201775x5.pdf]
