# Supplementary material for: APOBEC signature mutation generates an oncogenic enhancer that drives LMO1 expression in T-ALL
Source: Leukemia. 2017 Mar 28;31(10):2057–64. doi: 10.1038/leu.2017.75 (PMC5629363; doi:10.1038/leu.2017.75)

Figure S5: Monoallelic expression of *LMO1* in a primary T-ALL patient harbouring the C-to-T somatic enhancer mutation (indicated by the black arrow). Variant allele frequency (VAF) in tumour DNA and RNA of the 18 SNPs selected for assessing allelic imbalance in RNA-seq are shown at the top. All SNPs exhibit monoallelic expression of *LMO1* gene in RNA-seq. The expression level of *LMO1* is shown below as a wiggle plot.

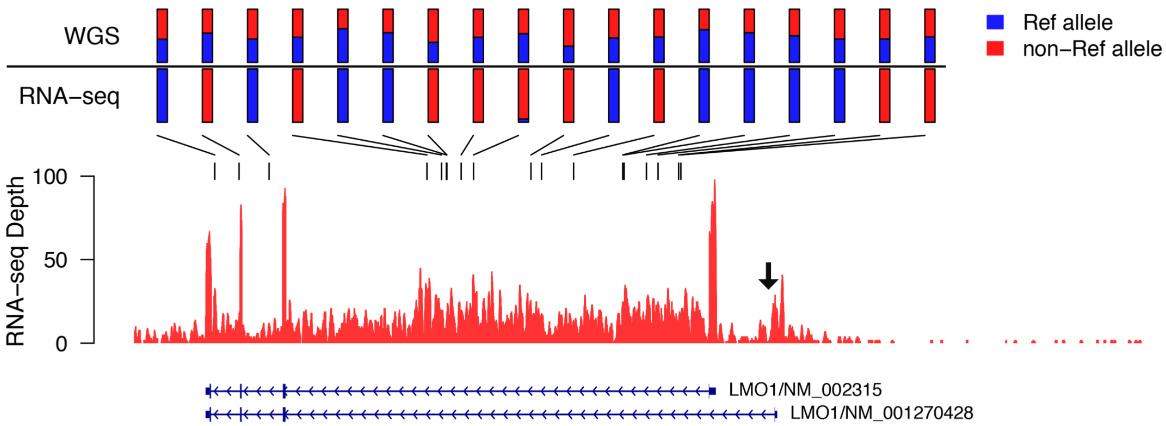

Supplement: Supplementary Figure 5 [file leu201775x6.pdf]
